# Complete mitochondrial genome sequences of two ground crickets, *Dianemobius fascipes nigrofasciatus* and *Polionemobius taprobanensis* (Orthoptera: Grylloidea: trigonidiidae)

**DOI:** 10.1080/23802359.2023.2285400

**Published:** 2023-12-11

**Authors:** Kohyoh Murata, Kosuke Kataoka, Ryuto Sanno, Kazuhiro Satomura, Atsushi Ogura, Toru Asahi, Kei Yura, Takeshi Suzuki

**Affiliations:** a Graduate School of Bio-Applications and Systems Engineering, Tokyo University of Agriculture and Technology, Tokyo, Japan; b Comprehensive Research Organization, Waseda University, Tokyo, Japan; c Graduate School of Advanced Science and Engineering, Waseda University, Tokyo, Japan; d Department of Bioscience, Nagahama Institute of Bio-Science and Technology, Shiga, Japan; e Global Consolidated Research Institute for Science Wisdom, Waseda University, Tokyo, Japan; f Institute for Advanced Research of Biosystem Dynamics, Waseda Research Institute for Science and Engineering, Waseda University, Tokyo, Japan; g Research Organization for Nano & Life Innovation, Waseda University, Tokyo, Japan; h Graduate School of Humanities and Sciences, Ochanomizu University, Tokyo, Japan; i Computational Bio Big-Data Open Innovation Laboratory (CBBD-OIL), National Institute of Advanced Industrial Science and Technology, Tokyo, Japan

**Keywords:** Complete mitogenome, crickets, phylogenetic analysis, speciation

## Abstract

The authors sequenced the complete mitochondrial (mt) genomes of the band-legged ground cricket (*Dianemobius fascipes nigrofasciatus* Matsumura, 1904) and a temperate form of the lawn ground cricket (*Polionemobius taprobanensis* Walker, 1869), collected in Japan. The length of the mt genome sequences was 15,354 bp in *D. fascipes nigrofasciatus* and 16,063 bp in *P. taprobanensis*. Annotation of the mt genome sequences revealed 13 protein-coding genes, two rRNA genes, and 22 tRNA genes. The orientation of the genes was the same as in other Grylloidea species, and the order was the same as in other Trigonidiidae species. In our phylogenetic analysis, *D. fascipes nigrofasciatus* formed a clade with *D. fascipes* collected in China, and the temperate form of *P. taprobanensis* formed a clade with *P. taprobanensis* collected in China. Comparison of the numbers of positions with different amino acid residues encoded by the protein-coding genes implied the separate species status of each member of each of the two pairs of ground crickets. The mt genome sequences of *D. fascipes nigrofasciatus* and *P. taprobanensis* will contribute to phylogenetic and taxonomic studies of the Trigonidiidae.

## Introduction

The band-legged ground cricket, *Dianemobius fascipes nigrofasciatus* (Matsumura 1904), is distributed in temperate regions such as southern Siberia, Northeast China, Korea, and Japan (Hokkaido, Honshu, Kyushu, Shikoku islands) (Benediktov and Storozhenko 2018). A subspecies, *Dianemobius fascipes fascipes* (Walker, 1869) is distributed in subtropical regions such as India, Sri Lanka, Nepal, Myanmar, Thailand, Vietnam, Malaysia, Indonesia, the Philippines, South and Southeast China, and Japan (Ryukyu Islands) (Benediktov and Storozhenko 2018). Taxonomically, *D. fascipes nigrofasciatus* has been confirmed to be a subspecies of *D. fascipes fascipes* (nominotypical subspecies) by differences in the acoustic calling signals, the color of the hind tibia, the shape of the ectoparamere lobe, and similarities in the male genital morphology (Benediktov and Storozhenko 2018). Male hybrids between temperate (= *D. fascipes nigrofasciatus*) and subtropical (= *D. fascipes fascipes*) forms are almost always sterile (Masaki 1983).

Contrary to the clear taxonomic status of the *Dianemobius* group, there has been controversy in the taxonomic status and notation of the lawn ground cricket, *Polionemobius taprobanensis* (Walker, 1869). As far as we know, the newest description concerning this classification was that *Polionemobius mikado* (Shiraki, 1911) is a synonym for a northern group of *P. taprobanensis* (He 2018). *Polionemobius taprobanensis* is distributed in Korea, Japan, Russia (far east), China, Taiwan, Southeast Asia, Indonesia, India, and Sri Lanka (Storozhenko et al. 2015). According to Masaki (1983), temperate (= northern) and subtropical (= southern) forms of the lawn ground cricket in Japan, with Tokunoshima (Ryukyu Islands) as the border of their distribution, were distinguished on the basis of differences in their ovipositor length, diapause, and photoperiodic responses. Masaki (1983) also argued that the names *Pteronemobius mikado* and *Pteronemobius taprobanensis* should be applied to the temperate and subtropical forms, respectively. However, Storozhenko et al. (2015) found no substantial differences in the shape of the male genitalia among specimens from Sri Lanka, India, Southeast Asia, China, Japan, Korea, and Russia. They further showed that the ovipositor length varied among females sampled in the same area. Masaki (1983) reported that hybrids of the temperate and subtropical forms and their F2 forms are fertile.

These ground crickets are often used as models of geographic variation (Masaki 1979a, 1979b, 1979c, 1983; Matsuda et al. 2018), diapausing (Masaki 1979a, 1983; Shiga and Numata 1997; Matsuda and Numata 2019), and photoperiodic response (Masaki 1979a, 1983). Interestingly, the distribution patterns are similar in the two pairs *D. fascipes fascipes*—*D. fascipes nigrofasciatus* and temperate—subtropical forms of *P. taprobanensis* (Masaki 1983). Furthermore, *D. fascipes nigrofasciatus* and the temperate form of *P. taprobanensis* exhibit photoperiod-dependent egg diapause, while *D. fascipes fascipes* and the subtropical form of *P. taprobanensis* have no diapause (Masaki 1983). The sequences of the mitochondrial (mt) genomes of *D. fascipes* with no subspecific names provided and *P. taprobanensis* of unknown form collected in China have already been registered (GenBank: MK303550.1 and NC_045848.1) (Ma et al. 2019b). Here, we determined the mt genome sequences of *D. fascipes nigrofasciatus* and a temperate form of *P. taprobanensis*, as well as their phylogenetic status.

## Materials and methods

**Figure 1.**
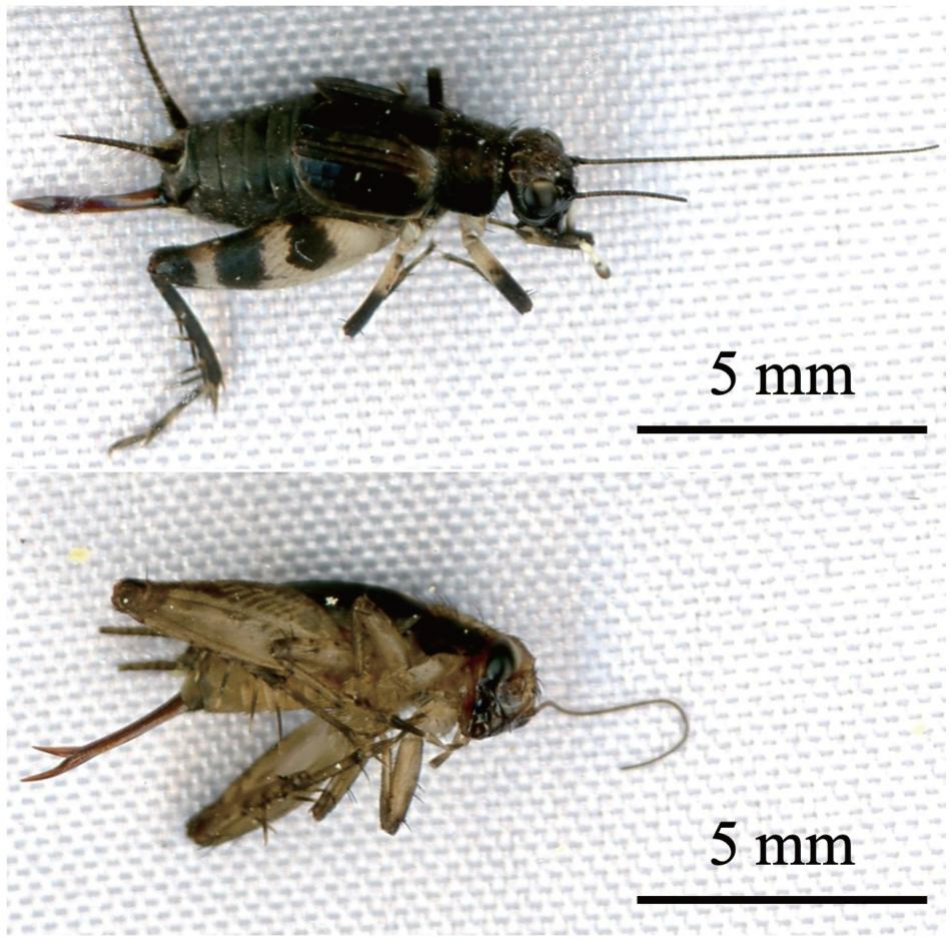
Images of adult specimens of (a) *Dianemobius fascipes nigrofasciatus* and (b) *Polionemobius taprobanensis* (temperate form). Photographs were taken by Kohyoh Murata. Original files of the photographs are made publicly available in figshare at https://doi.org/10.6084/m9.figshare.21781610.v3.

Adult specimens of *D. fascipes nigrofasciatus* and *P. taprobanensis* were collected from the ground at the Institute of Marine and Coastal Research of Ochanomizu University, Tateyama, Chiba, Japan (34.98°N, 139.82°E), under the permission by Prof. Masato Kiyomoto, the director of the facility. Specimens of *D. fascipes nigrofasciatus* (Figure 1) were distinguished from *D. fascipes fascipes* by the color patterns on the hind femurs and tibias (Benediktov and Storozhenko 2018). Specimens of *P. taprobanensis* were the temperate form distinguished from the subtropical form by the difference in ovipositor length. The length in *P. taprobanensis* of our specimen turned out to be 3.4 mm, as measured using NIH ImageJ (ver. 1.53k; Schneider et al. 2012), which corresponds to the length described in the temperate form by Masaki (1983). Genomic DNA of these two species was extracted using a QIAamp DNA Mini Kit (QIAGEN, Venlo, The Netherlands) in accordance with the manufacturer’s manual. Since genomic DNA was extracted from whole bodies of the samples after the specimen identification performed by Kohyoh Murata, no body parts were retained but original photographs of the specimens are stored as e-vouchers and openly accessible on figshare at https://doi.org/10.6084/m9.figshare.21781610.v3. Furthermore, other individuals of the same two species, which were collected in the same region and at the same time as the samples used in this study, are deposited as secondary specimens in the Center for Molecular Biodiversity Research, National Museum of Nature and Science (https://www.kahaku.go.jp/; 4-1-1 Amakubo, Tsukuba, Ibaraki 305-0005, Japan; Utsugi Jinbo, cmbr_cc@kahaku.go.jp), under voucher numbers NSMT-DNA 53743 and NSMT-DNA 53744 for *D. fascipes nigrofasciatus* and the temperate form of *P. taprobanensis*, respectively. The mt genomes were sequenced using the NovaSeq 6000 platform (Illumina, San Diego, California) and assembled using GetOrganelle (ver. 1.7.3.4-pre; Jin et al. 2020). According to the protocol previously described (https://www.protocols.io/view/generating-sequencing-depth-and-coverage-map-for-o-4r3l27jkxg1y/v1), we validated the assembly by mapping all raw reads to the mitochondrial genomes and evaluating the sequencing coverage depth (Fig. S1). Automatic annotation was performed by MITOS and MITOS2 web servers (Bernt et al. 2013; Donath et al. 2019). To compare protein-coding gene (PCG) sequences in the Grylloidea, mt genome data of another 17 Grylloidea species were downloaded from NCBI Genome at https://www.ncbi.nlm.nih.gov/genome/. Alignment of the PCG sequences using MEGAⅩ (ver. 10.1.8; Kumar et al. 2018) showed evident discordances in gene start and termination points compared with those of the other cricket species. The annotation was therefore manually curated. Phylogenetic analyses were performed using MEGAⅩ and were based on the sequences of all 13 PCGs of the 19 crickets; the disturbed regions in their alignment were removed using trimAl (ver. 1.4. rev15; Capella-Gutiérrez et al. 2009). We generated an initial phylogenetic tree using the neighbor-joining method (Saitou and Nei 1987) and then generated the final tree using the maximum likelihood method (Felsenstein 1981). To test the significance of branching in the tree, the bootstrap method was adopted for both analyses (Felsenstein 1985). For the analyses using neighbor joining, the Jones-Taylor-Thornton model (Jones et al. 1992) with gamma distribution adjustment was adopted, and for those using the maximum likelihood method, the mtREV model (Adachi and Hasegawa 1996) with adjustments of frequency, invariant sites, and gamma distribution was adopted, in accordance with an advanced model test in MEGAⅩ. Sequences of 13 PCGs of the *Drosophila melanogaster* subgroup (*Drosophila mauritiana* (NC_005779), *Drosophila simulans* (MN046104), *Drosophila sechellia* (MK659840), and *Drosophila melanogaster* (U37541)) were downloaded from NCBI to compare the numbers of positions with different amino acid residues between pairs of these species and the two pairs of crickets.

## Results

**Figure 2.**
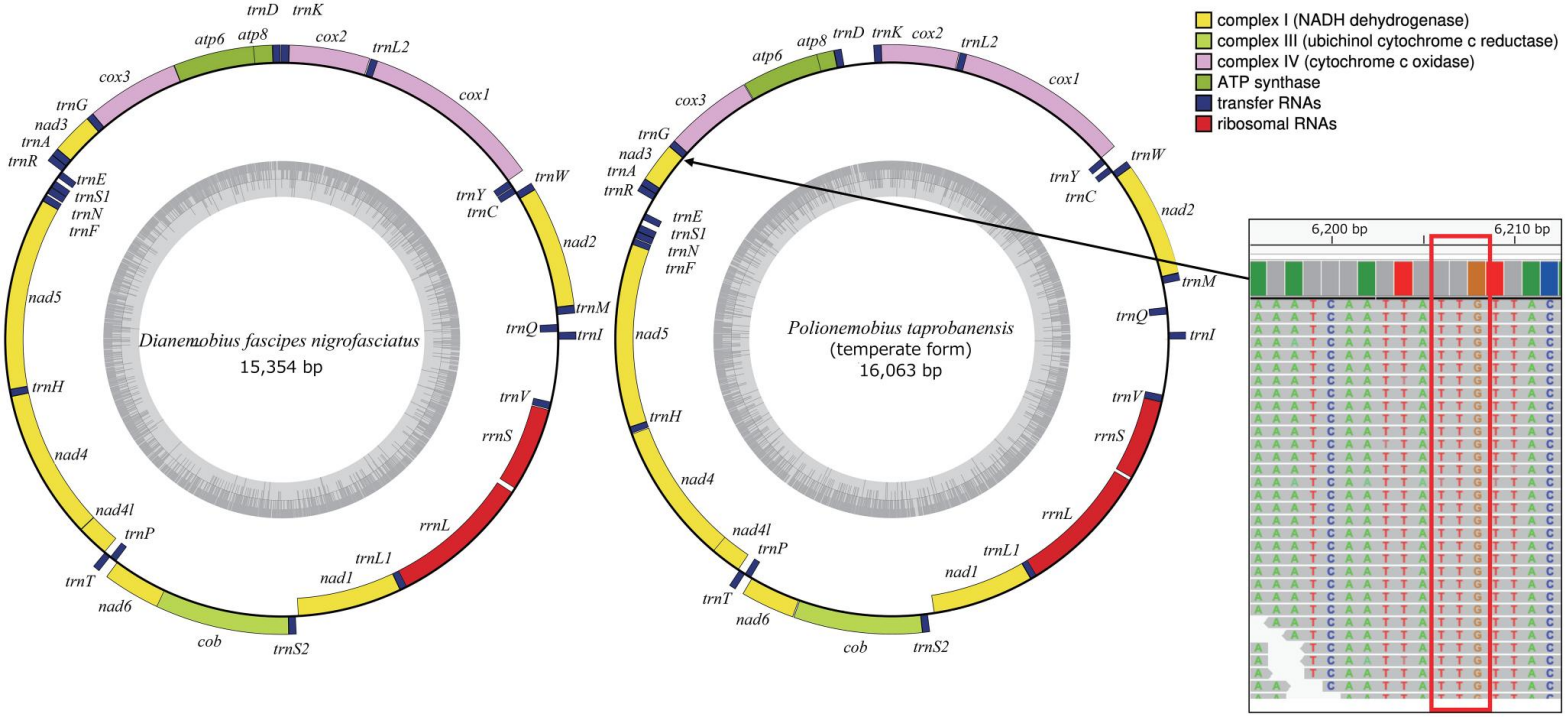
Maps of the mitochondrial genomes of *Dianemobius fascipes nigrofasciatus* and *Polionemobius taprobanensis* (temperate form). the outer and inner rings represent heavy and light chains, respectively. Different colors indicate different gene families. The darker and lighter gray area in the inner circle represent the GC and AT contents, respectively. Reads mapped at the TTG start codon (red frame, 6206–6208 nt) in *nad3* of the *P. taprobanensis* mitochondrial genome were also shown in right.

**Figure 3.**
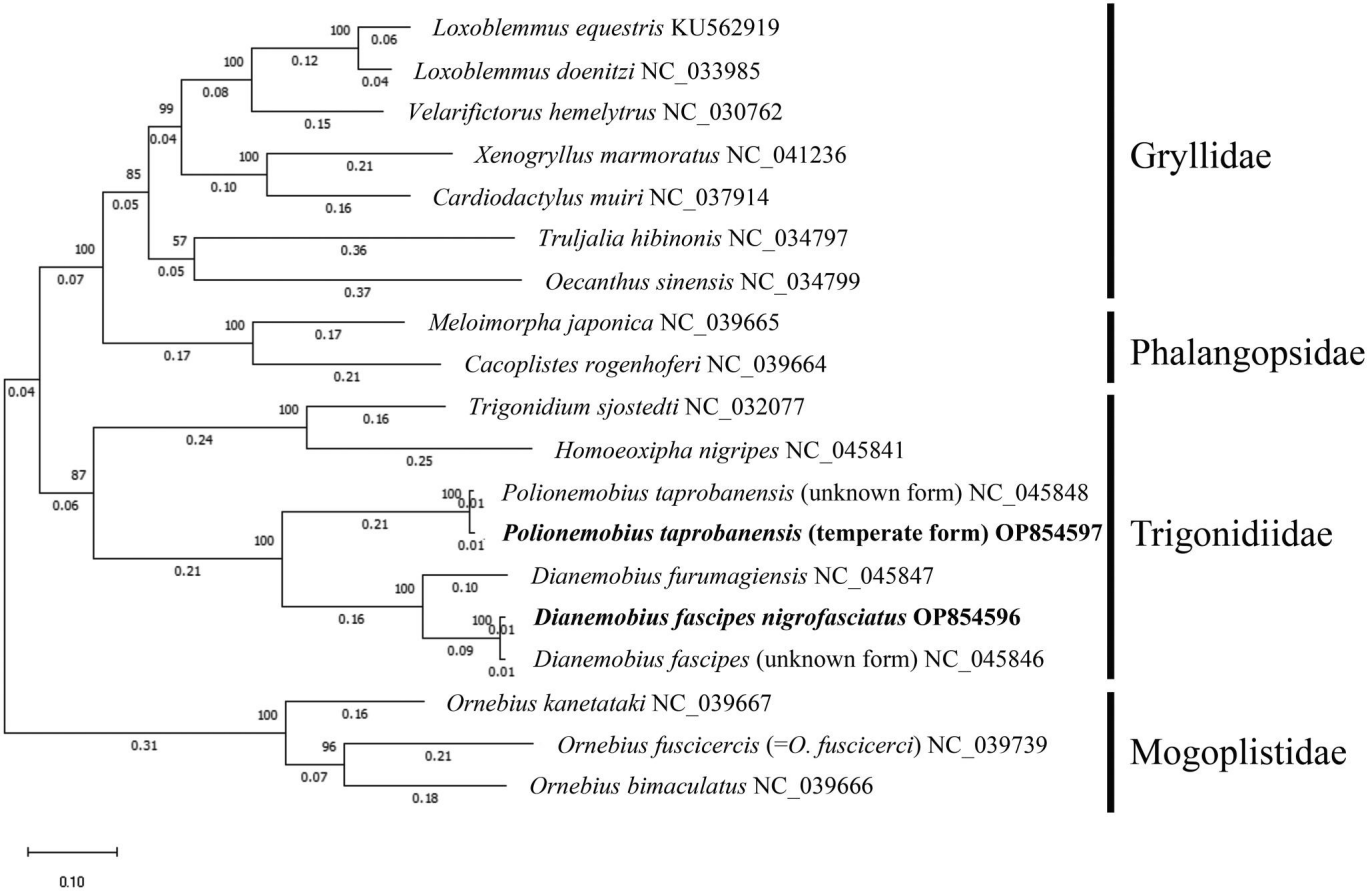
Maximum likelihood phylogenetic tree of 19 Grylloidea species with 1000 bootstraps. Bold text denotes species of which the sequences were newly revealed in this study. The amino acid sequences of the 13 PCGs of following species were also used: *L. equestris* KU562919 (Yang et al. 2016), *L. doenitzi* NC_033985 (unpublished), *V. hemelytrus* NC_030762 (Yang et al. 2016), *X. marmoratus* NC_041236 (Ma et al. 2019a), *C. muiri* NC_037914 (Dong et al. 2017), *T. hibinonis* NC_034797 (Li et al. 2019), *O. sinensis* NC_034799 (Li et al. 2019), *M. japonica* NC_039665 (Ma and Li 2018), *C. rogenhoferi* NC_039664 (Ma and Li 2018), *T. sjostedti* NC_032077 (Song et al. 2016), *H. nigripes* NC_045841 (Ma et al. 2019b), *P. taprobanensis* (unknown form) NC_045848 (Ma et al. 2019b), *P. taprobanensis* (temperate form) OP854597, *D. furumagiensis* NC_045847 (Ma et al. 2019b), *D. fascipes nigrofasciatus* OP854596, *D. fascipes* (unknown form) NC_045846 (Ma et al. 2019b), *O. kanetataki* NC_039667 (Ma and Li 2018), *O. fuscicercis* NC_039739 (Ma and Li 2018), and *O. bimaculatus* NC_039666 (Ma and Li 2018).

The length of the mt genome of *D. fascipes nigrofasciatus* was 15,354 bp, and that of the temperate form of *P. taprobanensis* was 16,063 bp (Figure 2). These included the 13 PCGs (*nad2*, *cox1*, *cox2*, *atp8*, *atp6*, *cox3*, *nad3*, *nad5*, *nad4*, *nad4L*, *nad6*, *cob*, and *nad1*), two rRNA genes (*rrnL* and *rrnS*), and 22 tRNA genes (*trnI*, *trnQ*, *trnM*, *trnW*, *trnC*, *trnY*, *trnL2*, *trnK*, *trnD*, *trnG*, *trnA*, *trnR*, *trnE*, *trnS1*, *trnN*, *trnF*, *trnH*, *trnT*, *trnP*, *trnS2*, *trnL1*, and *trnV*), of which the orientation was the same as in other Grylloidea species (Kataoka et al. 2020; Sanno et al. 2021). The gene order around *trnV* is *rrnL–rrnS–trnV* in Trigonidiidae species, although it is *rrnL–trnV–rrnS* in many other Grylloidea species (Ma et al. 2019b; Sanno et al. 2021). Gene order characteristic of the Trigonidiidae was observed in *D. fascipes nigrofasciatus* and the temperate form of *P. taprobanensis*. Non-canonical start codons were observed in *cox1* and *nad1* of *D. fascipes nigrofasciatus* and in *cox1*, *nad1*, and *nad3* of the temperate form of *P. taprobanensis*. Previous studies have shown the presence of non-canonical start codons in *cox1* and *nad1* in Orthopteran insects (Sheffield et al. 2010; Sanno et al. 2021), but none of the previous reports had found a TTG start codon in *nad3*. We checked the read coverage depth of this region (6206–6208 nt) and found that the TTG start codon in *nad3* was likely genuine (Figure 2). Our phylogenetic analysis confirmed that *D. fascipes nigrofasciatus* and *P. taprobanensis* belonged to the Trigonidiidae family (Figure 3). In addition to the Trigonidiidae, our phylogenetic tree generated three other clades—Gryllidae, Phalangopsidae, and Mogoplistidae—of which the topology corresponded to that in previous studies (Figure 3; Ma et al. 2019b; Sanno et al. 2021). In addition, *D. fascipes nigrofasciatus* and the temperate form of *P. taprobanensis* formed clades with *D. fascipes* with no subspecific names provided and *P. taprobanensis* of unknown form collected in China, respectively. The branch length from the divergence of *D. fascipes* collected in China and *D. fascipes nigrofasciatus* was almost the same as that of *P. taprobanensis* collected in China and the temperate form (Figure 3). We found that the number of positions with different amino acid types encoded by the PCGs between *D. fascipes* collected in China and *D. fascipes nigrofasciatus* was 53, and that between *P. taprobanensis* collected in China and the temperate form was 49; these values were in the range of the differences between pairs of the four sister species of the *D. melanogaster* subgroup (Table 1).

**Table 1.** Numbers of amino acid differences in the 13 protein-coding genes.

Trigonidiidae		
	*D. fascipes* (unknown form)*—D. fascipes nigrofasciatus*	53
	*P. taprobanensis* (unknown form)*—P. taprobanensis* (temperate form)	49
*Drosophila melanogaster* subgroup		
	*D. simulans—D. mauritiana*	36
	*D. sechellia—D. mauritiana*	65
	*D. melanogaster—D. mauritiana*	99

## Discussion

These findings suggest that, within each pair, *D. fascipes* collected in China—*D. fascipes nigrofasciatus* collected in Japan and *P. taprobanensis* collected in China*—*the temperate form collected in Japan, are closely related, but different species. Each pair can be more easily determined by the *cox1* sequence; the former pair differed by 12 nt in the nucleotides from 1006 to 1368 and the latter pair differed by 7 nt in the nucleotides from 1204 to 1436. It is unclear whether the two crickets collected in China (Ma et al. 2019b) correspond to *D. fascipes fascipes* and the subtropical form of *P. taprobanensis*, respectively. In Japan, the members of each pair of *D. fascipes fascipes*—*D. fascipes nigrofasciatus* and temperate—subtropical forms of *P. taprobanensis* diverged by allopatric speciation, because their distributions are divided at about 25°N to 28°N in the Ryukyu Islands (Masaki 1983). Further studies, such as phylogenetic analyses by using mt genomes including the subtropical species, whole genomes, or population genetics, are required to reveal the details of these speciations.

## Conclusion

In summary, we revealed the complete mt genomes of two Trigonidiidae crickets, *D. fascipes nigrofasciatus* and the temperate form of *P. taprobanensis* collected in Japan, and found by phylogenetic analysis that they were closely related to *D. fascipes* and *P. taprobanensis* collected in China, respectively. The PCGs in the mt genomes of the two pairs of crickets seemed to encode proteins with enough amino acid differences for each member of each pair to be considered as a different species. These divergences are likely examples of allopatric speciation because of the division in the species’ distribution areas. Crickets have been used as models for studying speciation (Kataoka et al. 2022). The Trigonidiidae mt genome sequences revealed here will contribute to phylogenetic and taxonomic studies of crickets to elucidate the mechanisms of speciation.

## Supplementary Material

Supplemental Material

## Data Availability

The genome sequence data that support the findings of this study are openly available in GenBank of NCBI at https://www.ncbi.nlm.nih.gov/under the accession nos. OP854596 (*D. fascipes nigrofasciatus*) and OP854597 (temperate form of *P. taprobanensis*). The associated BioProject, SRA, and Bio-Sample numbers are PRJNA926495 (*D. fascipes nigrofasciatus* and temperate form of *P. taprobanensis*), SRX19142015 (*D. fascipes nigrofasciatus*) and SRX19142016 (temperate form of *P. taprobanensis*), and SAMN32875128 (*D. fascipes nigrofasciatus*) and SAMN32875129 (temperate form of *P. taprobanensis*), respectively.
