# Supplementary material for: Complete mitochondrial genome sequences of two ground crickets, Dianemobius fascipes nigrofasciatus and Polionemobius taprobanensis (Orthoptera: Grylloidea: trigonidiidae)
Source: Mitochondrial DNA B Resour. 2023 Dec 11;8(12):1311–5. doi: 10.1080/23802359.2023.2285400 (PMC10763891; doi:10.1080/23802359.2023.2285400)
Supplement: Supplemental Material [file TMDN_A_2285400_SM4959.pdf]

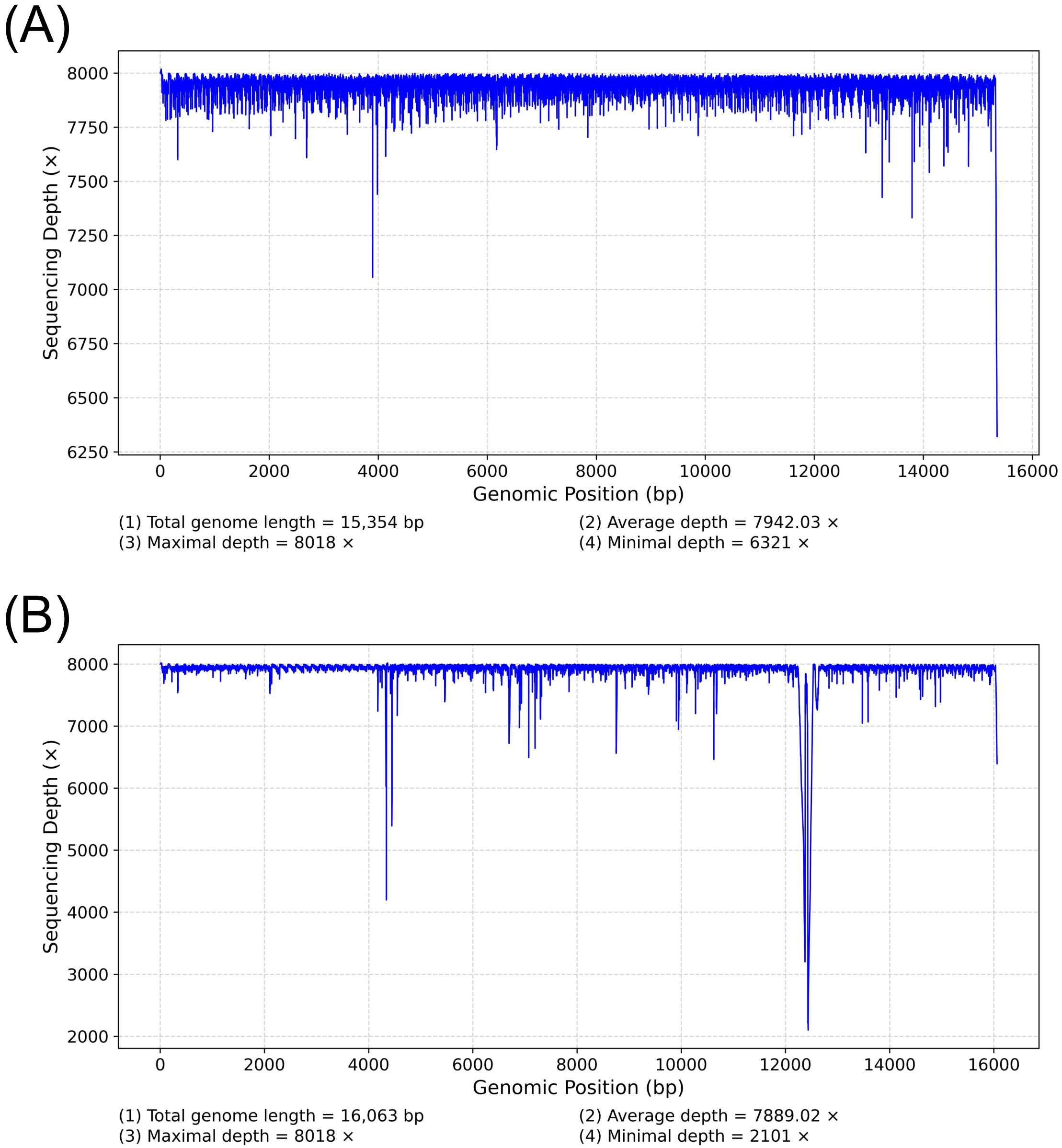

Fig. S1. Mt genome sequencing coverage maps of (A) *Dianemobius fascipes nigrofasciatus* and (B) *Polionemobius taprobanensis* (temperate form).
